# Measuring health-related social deprivation in small areas: development of an index and examination of its association with cancer mortality

**DOI:** 10.1186/s12939-021-01545-9

**Published:** 2021-09-27

**Authors:** Kailu Wang, Chi-Kin Law, Jiaying Zhao, Alvin Yik-Kiu Hui, Benjamin Hon-Kei Yip, Eng Kiong Yeoh, Roger Yat-Nork Chung

**Affiliations:** 1 JC School of Public Health and Primary Care, Faculty of Medicine , The Chinese University of Hong Kong, Shatin, NT, Hong Kong SAR, China; 2 Centre for Health Systems and Policy Research, Faculty of Medicine, The Chinese University of Hong Kong, Shatin, NT, Hong Kong SAR, China; 3 NHMRC Clinical Trials Centre, University of Sydney, Camperdown, NSW Australia; 4 School of Demography, The Australian National University, Canberra, Australia; 5 CUHK Institute of Health Equity, The Chinese University of Hong Kong, Shatin, NT, Hong Kong SAR, China

**Keywords:** Deprivation, Inequalities, Chronic disease, Cancer, Small area indices

## Abstract

**Background:**

The small-area deprivation indices are varied across countries due to different social context and data availability. Due to lack of chronic disease-related social deprivation index (SDI) in Hong Kong, China, this study aimed to develop a new SDI and examine its association with cancer mortality.

**Methods:**

A total of 14 socio-economic variables of 154 large Tertiary Planning Unit groups (LTPUGs) in Hong Kong were obtained from 2016 population by-census. LTPUG-specific all-cause and chronic condition-related mortality and chronic condition inpatient episodes were calculated as health outcomes. Association of socio-economic variables with health outcomes was estimated for variable selection. Candidates for SDI were constructed with selected socio-economic variables and tested for criterion validity using health outcomes. Ecological association between the selected SDI and cancer mortality were examined using zero-inflated negative binomial regression.

**Results:**

A chronic disease-related SDI constructed by six area-level socio-economic variables was selected based on its criterion validity with health outcomes in Hong Kong. It was found that social deprivation was associated with higher cancer mortality during 2011–2016 (most deprived areas: incidence relative risk [IRR] = 1.40, 95% confidence interval [CI]: 1.27–1.55; second most deprived areas: IRR = 1.34, 95%CI: 1.21–1.48; least deprived areas as reference), and the cancer mortality gap became larger in more recent years. Excess cancer death related to social deprivation was found to have increased through 2011–2016.

**Conclusions:**

Our newly developed SDI is a valid and routinely available measurement of social deprivation in small areas and is useful in resource allocation and policy-making for public health purpose in communities. There is a potential large improvement in cancer mortality by offering relevant policies and interventions to reduce health-related deprivation. Further studies can be done to design strategies to reduce the expanding health inequalities between more and less deprived areas.

**Supplementary Information:**

The online version contains supplementary material available at 10.1186/s12939-021-01545-9.

## Introduction

Social deprivation is a concept referring to disadvantages of an individual or a group of individuals in accessing material and social resources and fragility of social networks from family to community [1, 2]. Plenty of aspects need to be involved to comprehensively define social deprivation and therefore, selection of indicators to construct an index for measuring social deprivation should consider a number of demographic, social, and economic domains. Studies have found socio-economic domains that are related to social deprivation, including income, education, home ownership, employment status, and social class in terms of occupation [2–4]. Family-related factors, such as marital status, family size, and structure, were also considered to be relevant indicators to social deprivation on both area level and individual level [5–7]. Several indices developed in 1980s, including the Underprivileged Area (UPA) score [4], the Carstair score [3], the Townsend score [2], and numerous indices in more recent years in Europe [8, 9], North America [10, 11] and New Zealand [12], provided a wide range of selection for variables for constructing social deprivation index. The various measurement of social deprivation in different countries reflected that no universal rule can be applied to define social deprivation worldwide as it is varied and context-specific.

The deprivation indices have been used to understand health inequalities since 1980s [3]. In recent years, evidence was found that the disparities of premature mortality and morbidity became larger between affluent and deprived population groups or areas. Studies in the US [13, 14] have found a widening gap in all-cause mortality and life expectancy between more and less deprived areas among all socio-economic and ethnic subgroups. While studies have been widely conducted for inequalities in all-cause, cardiovascular disease, injury-related morbidity and mortality [15, 16], inequalities in cancer were receiving relatively less but growing attention. A population-based study over more than a decade also found emerging inequalities in colorectal cancer incidence and persistent inequalities in lung cancer [17]. Expanding contribution of cancer to income inequalities in all-cause mortality was found in a nationwide study in New Zealand [18]. The difference in survival between high-income and low-income cancer patients was also found to be larger in more recent years [19]. However, these studies mainly came from the Western countries. Evidence is insufficient about whether the effect of deprivation or lower income on cancer incidence, mortality, or survival in other non-Western countries and regions has a similar pattern. A recent study in South Korea found a decreasing survival time of patients with cancer in more deprived areas [20], and another study from the same country used a deprivation index as a covariate in finding association between environmental pollution and a few types of cancer [21], highlighting a need for more studies in different countries and regions in Asia.

Hong Kong, a special administrative region of China, is categorized as a high-income economy by the World Bank [22]. However, there is a substantial disparity in household income, as Gini coefficient was reported to be 0.539 in 2016, while a coefficient higher than 0.5 is usually considered to indicate severe disparity [23]. A widening income disparity was also observed in Hong Kong as the Gini coefficient increased from 0.518 to 0.539 from 1996 to 2016 [23]. Meanwhile, Hong Kong is facing the problem of population ageing in recent years. People aged 65 and above comprised 16% of the population in Hong Kong, and this percentage was estimated to reach 23% in 2026 [24]. Around 78% of people aged over 65 suffered from chronic diseases in 2019, and the most updated statistics in 2009 showed that over 40% of older persons had more than one chronic condition [25, 26]. With an ageing population and widening income disparities in Hong Kong, it is anticipated that the burden for public healthcare system would become heavier, and poorer and more deprived persons could be vulnerable in getting timely access to adequate healthcare services for chronic diseases, including cancer. Moreover, cancer is the second highest cause of death worldwide [27] and the highest in Hong Kong [28]. Therefore, it is necessary to understand how deprivation influences the burden of cancer.

In Hong Kong, an individual-level deprivation index was developed and its associations with various health outcomes were also found [29–32]. Nevertheless, the construction of this index requires the administration of individual-based survey, which may not be always feasible. On the other hand, making use of routine government data, two area-level social deprivation indices were developed for suicide risk [33] and environmental risk on mortality [34], respectively. Nevertheless, while Hsu et al. [33] reported comprehensive findings on association between suicide and socio-economic inequalities, some of the variables used for constructing their index were not readily available in current routine databases. Considering this, Hsu et al.’s index cannot be directly compared with other indices in examining the association between chronic conditions and deprivation. For the index used in Wong et al.’s study [34], its major purpose was to examine the impact of air pollution on the mortality in areas with different deprivation levels, so it was not designed to explore the magnitude of inequalities in chronic disease burden, which will be useful in health policy-making and planning for disease prevention and resource allocation, especially when individual-level data is limited; therefore, it is necessary to review the validity of this index and compare it with potential alternatives using updated data, and explore whether it is applicable to other health issues. The present study aims to validate the social deprivation index based on its more recent association with chronic disease morbidity and mortality, and update the index with more recent data to explore the association of deprivation with cancer mortality.

## Methods

The development and validation of a social deprivation index (SDI) comprise development and comparison of various SDIs that were constructed using different combinations of demographic and socio-economic variables with the index developed by Wong et al. [34] (referred to as “the previous SDI” below), which has been used to measure the level of deprivation in a number of studies in recent years [35–39]. We chose to take reference from Wong et al.’s index not only for its relevance to the local Hong Kong context, but also because it provided a comprehensive, multifaceted set of socio-economic indicators as candidates for constructing the SDI. A selected SDI based on criterion validity was then used to examine the association between social deprivation and cancer mortality. Ethical approval was granted by the appropriate ethics committee prior to commencement of the study.

### Data source and variable selection

Data comprised of socio-economic characteristics and health-related outcomes. Socio-economic characteristics of different large Tertiary Planning Unit groups (LTPUGs) for construction of the SDIs were obtained from the 2016 population by-census data [40]. In 2016, Hong Kong was divided into 154 LTPUGs, the small geographical areas with population size ranging from 10,691 to 286,232 that were determined by the Planning Department of Hong Kong Government for town planning purposes and used in conjunction with population census. A total of 14 socio-economic variables (Tables 1 and 2) that fall into eight domains representing the characteristics of population or households in different LTPUGs in census and by-census of Hong Kong were identified as factors contributing to social deprivation in previous studies [1, 34, 41], and were also used to construct the previous SDI by Wong et al. [34]. Age, sex, death time, cause of death using International Classification of Diseases (ICD)-10 coding, and residential area of deceased individuals in Hong Kong during 2006–2016 were obtained from the micro-dataset of Known Death from Census and Statistics Department. Mortality indicators to be tested included all-cause mortality under 75 years and chronic condition-related mortality under 75 years. Morbidity indicator was chronic condition-related inpatient episode, which was derived from the 2006–2015 health records of the Hospital Authority, the statutory body managing all public hospitals in Hong Kong. The data during 2006–2010 was used to develop and validate the SDI, and the data during 2011–2016 was used to examine the association between the SDI and cancer mortality. Those with missing data in residential area (i.e. coding of LTPUGs) were removed. There were no missing data in the datasets used for calculating mortality indicators, and the pattern of missing data in the dataset for morbidity indicators (i.e. inpatient episode) can be found in Supplementary file (Table S2). Chronic conditions selected for the analysis included hypertension, diabetes mellitus, high cholesterol, heart diseases, asthma, chronic obstructive pulmonary disease (COPD), and stroke or cerebrovascular diseases (see ICD-10 codes in supplementary file), which together make up 60% of prevalence of chronic conditions in Hong Kong [42].

**Table 1:** Percentage distribution of individual/household with certain socio-economic characteristics in large Tertiary Planning Unit groups

	N	Mean	Standard deviation	Minimum	25 percentile	Median	75 percentile	Maximum
No schooling	154	19.58%	3.99%	11.17%	16.71%	19.41%	22.18%	32.58%
Degree course	154	30.08%	10.37%	12.32%	22.26%	27.98%	36.59%	55.35%
Low income	154	18.68%	5.42%	6.00%	14.90%	18.79%	22.03%	43.02%
Unemployment	154	4.54%	1.41%	0.92%	3.52%	4.64%	5.70%	8.13%
Managerial occupation	154	12.09%	6.64%	1.92%	7.19%	10.51%	16.50%	32.22%
Elementary occupation	154	22.18%	5.50%	14.20%	18.99%	21.01%	23.69%	49.73%
Divorced/separated	154	5.03%	1.28%	2.68%	4.24%	4.84%	5.64%	11.13%
Never-married	154	37.79%	2.16%	31.42%	36.40%	37.71%	39.40%	44.33%
Nuclear family	154	63.10%	7.00%	44.25%	58.23%	64.30%	67.57%	78.99%
One-person household	154	18.80%	6.53%	4.54%	14.61%	17.20%	23.00%	39.32%
Two-persons household	154	26.33%	3.98%	11.68%	24.23%	26.84%	28.90%	36.65%
6 + persons household	154	4.26%	3.37%	0.37%	2.28%	3.27%	4.68%	23.48%
Ownership	154	51.38%	17.07%	0.07%	40.88%	53.84%	62.83%	82.92%
Sub-tenancy	99^a^	0.40%	0.41%	0.01%	0.12%	0.21%	0.61%	2.21%

^a^Data of sub-tenancy were missing in 55 LTPUGs

**Table 2:** Factor analysis of the 14 socio-economic variables from eight domains

Domains	Variable	Factor 1 (f1)	Factor 2 (f2)	Factor 3 (f3)
Education	no schooling	**0.71**	-0.38	0.01
	degree course	**-0.88**	0.41	-0.02
Income	low income	**0.79**	-0.16	0.16
Employment	unemployment	**0.74**	-0.33	0.01
Occupation	managerial occupation	**-0.87**	0.28	0.02
	elementary occupation	-0.48	-0.19	**0.71**
Marital status	divorced/separated	**0.62**	0.05	0.36
	never-married	0.19	0.26	0.32
Family composition	nuclear family	**-0.55**	**-0.64**	-0.27
Family size	one-person household	**0.51**	**0.80**	0.17
	two-persons household	**0.57**	0.26	-0.35
	6 + persons household	**-0.74**	-0.19	**0.52**
Tenure of accommodation	ownership	**-0.57**	0.12	-0.46
	sub-tenancy	0.20	0.41	0.02
Eigenvalue		6.05	2.00	1.58
Proportion		50.9%	16.9%	13.3%

### Construction of SDIs

The previous SDI by Wong et al. [34] was constructed using simple summation of the area-level proportion of six socio-economic factors: unemployment, monthly household income < HK$2000 (~ US$250), no schooling, one-person household, never married persons, and sub-tenancy among each LTPUG. The higher the proportion of these factors, the higher the SDI; and the higher the SDI, the more deprived the area is. Among these six variables, the cut-off of monthly household income used in the previous SDI was an absolute value and was out of date; thus, 50% of the median monthly domestic household income by household size was used instead as the new threshold to define low income household in this study as it has been used as the official poverty line in Hong Kong to indicate “relative poverty” since 2012 [43]. The new SDIs were derived from the 14 socio-economic variables as mentioned above using simple summation of their proportions or factor scores calculated using factor analysis.

### Statistical analysis

Age- and sex-standardized inpatient episode rate, average annual premature all-cause mortality (under age 75), and average annual premature chronic condition-related mortality (under age 75) during 2006–2010 were calculated for each of the LTPUGs as outcomes of the analysis. Socio-economic characteristics of the 154 LTPUGs in terms of distribution of the 14 socio-economic variables were described. Factor analysis was performed on the 14 socio-economic variables to examine which of these variables were representative indicators of social deprivation in small areas. Univariate linear regressions were then used to determine the association between health outcomes during 2006–2010 and these socio-economic variables, in order to select potential variables for the construction of the SDIs. Different sets of variables were derived from the univariate analysis to construct different SDIs. Candidate SDIs were constructed based on results of the univariate regressions, and selection of the one set of variables to best represent SDI was based on the criterion validity of these SDIs as tested by Pearson’s correlation. The selected continuous index was recoded as categorical measures using quartiles as cut-offs to reduce uncertainty in measurement. The Spearman correlations between health outcomes and the SDI categories during 2011–2016 were then used to further test the external validity of the SDI categories.

The selected SDI was used to examine the ecological association between social deprivation and cancer mortality using zero-inflated negative binomial regression with adjustment of age, sex, year, and population size, as it was found to have better goodness of fit in modeling over-dispersion data with excess zeros than standard negative binomial regression and zero-inflated Poisson regression [44]. Age-, sex-, LTPUG- and calendar-year-specific cancer death frequency and population were also calculated separately. Each observation in the dataset contains the cancer death count of a specific age and sex group in a specific LTPUG in a specific year. LTUPGs were divided into four groups based on their levels of social deprivation — Group 1 refers to the least deprived with the SDI value below 25th percentile; Group 2’s SDI lies between 25 and 50th percentile; Group 3’s SDI lies between 50 and 75th percentile; and Group 4’s SDI refers to the most deprived where SDI lies above 75th percentile. Group 1 was used as the reference category in the regression. The interaction effects between social deprivation and calendar years on cancer-caused death were tested. All analyses were conducted in Stata 14 and 15, Windows version (StataCorp, College Station, Texas 77845 USA).

## Results

### Representativeness of the 14 socio-economic variables

Distributions of percentage of individuals or households with certain socio-economic characteristics in 154 LTPUGs are shown in Table 1. Socio-economic and health data of 154 LTPUGs were retrieved and compiled for analysis. In the factor analysis for the 14 socio-economic variables, three factors (Factor 1–3, marked as f1, f2 and f3 in Table 2) were identified and each of them explained at least 10% of the variance, accounting for 81.1% of the variation in total. F1 explained the greatest of the variation (50.9%), representing LTUPGs with higher proportions of people being unemployed and being divorced, and with lower education level and lower income. f2 represented LTPUGs with more one-person households, and f3 represented those with more people living with a relatively big family (over six persons) and working in elementary occupations. Among the 14 variables, never married and sub-tenancy did not have a factor loading higher than 0.5 in any of the three factors.

### Association of socio-economic variables with health outcomes

The associations of the 14 socio-economic variables with the three health outcomes were determined separately using univariate regressions (Table 3). Most socio-economic variables were associated with chronic condition-related inpatient episode rate. For premature all-cause mortality, it was only associated with no schooling, low income, nuclear family, two-person household, and ownership of accommodation. Only sub-tenancy was not associated with any of the three health outcomes. At least one variable was selected for the construction of the SDI from each socio-economic category to ensure the SDI covered all aspects of socio-economic characteristics. Therefore, no schooling (education), low income (income), managerial occupation (employment and occupation), divorced/separated (marital status), nuclear family (family composition), one-person household or two-person household (family size), and ownership (tenure of accommodation) were selected based on the result of the univariate regressions. Never married and sub-tenancy, which were factors in the previous SDI, were not included in our new SDI as they were found to have weaker association with health outcomes and lower representativeness of socio-economic characteristics according to our factor analysis.

**Table 3:** Univariate association between socio-economic variables and health outcomes during 2006–2010

Domains	Variables^a^ (The proportion of people with each of these status within an area)	Standardized episode rate^a^ (Inpatient)		Premature mortality (chronic condition-related)^a^		Premature mortality (All cause)^a^	
		Coef	*P* > t	Coef	*P* > t	Coef	*P* > t
Education	no schooling	1.37	< 0.001*	1.02	< 0.001*	0.87	0.014*
	degree course	-1.20	< 0.001*	-0.70	< 0.001*	-0.41	0.051
Income	low income	1.38	< 0.001*	1.13	< 0.001*	0.95	< 0.001*
Employment and occupation	unemployment	1.22	< 0.001*	0.25	0.129	0.03	0.890
	managerial occupation	-0.84	< 0.001*	-0.48	< 0.001*	-0.22	0.075
	elementary occupation	-0.93	0.007*	0.35	0.193	0.12	0.710
Marital status	divorced/separated	1.21	< 0.001*	0.80	0.001*	0.05	0.860
	never-married	3.15	0.022*	2.16	0.043*	1.81	0.160
Family composition	nuclear family	-1.07	0.108	-2.11	< 0.001*	-1.64	0.007*
Family size	one-person household	0.47	0.021*	0.43	0.008*	0.23	0.238
	two-persons household	2.29	< 0.001*	1.39	< 0.001*	0.89	0.039*
	6 + persons household	-0.80	< 0.001*	-0.31	0.001*	-0.05	0.639
Tenure of accommodation	ownership	-0.30	0.004*	-0.17	0.041*	0.28	0.001*
	sub-tenancy	-0.11	0.242	0.03	0.627	0.04	0.546

^*^P < 0.05

^a^These indicators were transformed into logarithm form in regression

### Criterion validity of SDIs

Six candidate sets of SDI were constructed for further examination (Table 4). The Pearson correlation (r) indicated that SDI6 had stronger correlation with health outcomes than all the other five candidate sets, including the previous one by Wong et al. [34] (Table 4). Therefore, SDI6 had a better criterion validity than other SDI candidates. Further tests with inpatient episode rate and premature mortality during 2011–2016 also showed that SDI6 had good external criterion validity in terms of Spearman’s rho that ranged from 0.39–0.58 across different years (Table 5).

**Table 4:** Pearson correlation between social deprivation index candidates and health outcomes during 2006–2010 and socio-economic factors

Construction of the SDI	SDI1	SDI2	SDI3	SDI4	SDI5	SDI6
	Previous SDI by Wong et al. (2008)^a^	Factor score of factor 1 from factor analysis of the six variables in SDI1	Factor score sum of factor 1–3 in factor analysis of the six variables in SDI1	Sum: no schooling, low income, non-nuclear family, divorced/separate, one-person household, and non-owner	Sum: no schooling, low income, non-nuclear family, divorced/separate, one-person household, and non-managerial occupation	Sum: no schooling, low income, non-nuclear family, divorced/separate, two-person household, and non-managerial occupation
SMR^b^ < 75 years (chronic diseases)	0.39*	0.31*	0.45*	0.39*	0.44*	0.45*
*P*-value	< 0.0001	0.0024	< 0.0001	< 0.0001	< 0.0001	< 0.0001
SMR^b^ < 75 years (All cause)	0.39*	0.33*	0.46*	0.35*	0.44*	0.46*
*P*-value	< 0.0001	0.0011	< 0.0001	< 0.0001	< 0.0001	< 0.0001
SER^c^ (Inpatient)	0.36*	0.40*	0.33*	0.39*	0.40*	0.48*
*P*-value	< 0.0001	0.0002	0.0027	< 0.0001	< 0.0001	< 0.0001
f1^d^ of 14-variables factor analysis	0.90*	0.81*	0.89*	0.82*	0.92*	0.95*
*P*-value	< 0.0001	< 0.0001	< 0.0001	< 0.0001	< 0.0001	< 0.0001
f2^d^ of 14-variables factor analysis	0.23*	-0.42*	-0.01	0.18*	0.25*	0.07
*P*-value	0.0034	< 0.0001	0.9329	0.0232	0.0021	0.4159
f3^d^ of 14-variables factor analysis	0.22*	0.26*	0.41*	0.43*	0.20*	0.09
*P*-value	0.0064	0.0085	< 0.0001	< 0.0001	0.014	0.2721

^*^P < 0.05; The value in the table are Pearson’s r and their *P* values

^a^Six variables for previous SDI by Wong et al. (2008): (1) unemployment, (2) low income, (3) no schooling, (4) one-person household, (5) never married persons, and (6) sub-tenancy

^b^*SMR* Standardized mortality rate

^c^*SER* Standardized episode rate

^d^They are factor scores from factor analysis of the 14 socio-economic variables, please refer to the Table 2

**Table 5:** Spearman correlation between selected social deprivation index in categorical form and health outcomes during 2006–2016

	SER^a^ (Inpatient)	SMR^b^ < 75 (Chronic disease)	SMR^b^ < 75 (All cause)
06–10 aggregate	0.50	0.56	0.55
*P* value	< 0.0001	< 0.0001	< 0.0001
2011	0.40	0.58	0.56
*P* value	< 0.0001	< 0.0001	< 0.0001
2012	0.39	0.45	0.53
*P* value	< 0.0001	< 0.0001	< 0.0001
2013	0.39	0.52	0.52
*P* value	< 0.0001	< 0.0001	< 0.0001
2014	0.40	0.46	0.46
*P* value	< 0.0001	< 0.0001	< 0.0001
2015	0.40	0.47	0.43
*P* value	< 0.0001	< 0.0001	< 0.0001
2016	-	0.39	0.39
*P* value	-	< 0.0001	< 0.0001

The values in the table are Spearman’s rho and their *P* values

^a^*SER* Standardized episode rate

^b^*SMR* Standardized mortality rate

### Association between social deprivation and cancer mortality

**Fig. 1:**
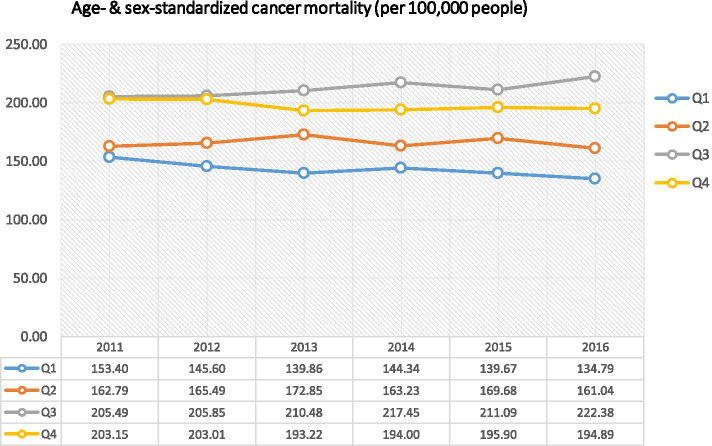
Age- and sex-standardized cancer mortality by areas in four SDI quartiles. Note: 1. SDI: social deprivation index. 2. The mortality rate was standardized based on the age and sex distribution of population in Hong Kong in 2016

In the logistic model, the LTPUG with younger population and fewer females tended to have zero death count of cancer death. The likelihood of having zero cancer death count increased through 2011–2016. The geographical variation of social deprivation level and cancer mortality are shown in Supplementary file (Figures S5 and S6). In the negative binomial model, risk of cancer death of Group 4 LTPUGs (the 25% most deprived) was around 40% higher than that in Group 1 (the 25% least deprived) (Table 6). Group 3 was also 34% more likely to die from cancer as compared with Group 1. Age- and sex-adjusted cancer mortality rate was increasing in Group 3, while it was decreasing in Group 1 from 2011 to 2016 (Table 7 and Fig. 1), and excess death due to social deprivation in areas of Group 2–4 also increased during the same period (Table 7). The increasing excess death and significant coefficient of interaction between Group 3 and calendar year of 2016 indicated a larger relative risk of cancer death between this group and the least deprived area in 2016 than in 2011.

**Table 6:** Zero-inflated negative binomial model for association of social deprivation with cancer mortality

		Logit model (likelihood of having zero death count)		Negative binomial model (relative risk of cancer mortality)	
		Adjusted OR	95% confidence interval	IRR^a^	95% confidence interval
**Year (2011 as reference)**					
2012		1.87	(0.48, 7.27)	1.01	(0.90, 1.14)
2013		3.30	(0.91, 12.00)	1.03	(0.92, 1.16)
2014		3.43	(0.94, 12.43)	1.08	(0.96, 1.22)
2015		1.74	(0.44, 6.91)	1.02	(0.91, 1.15)
2016		1.73	(0.41, 7.34)	0.99	(0.88, 1.12)
**Social deprivation level (Group 1 (least deprived) as reference)**					
Group 2		0.30	(0.05, 1.88)	1.05	(0.95, 1.16)
Group 3		**-**^b^	**-**	1.34*	**(1.21, 1.48)**
Group 4 (most deprived)		1.25	(0.35, 4.43)	1.40*	**(1.27, 1.55)**
**Interaction between social deprivation level and year**					
2012xGroup 2		-^b^	-	1.02	(0.89, 1.17)
2012xGroup 3		-^b^	-	0.98	(0.86, 1.13)
2012xGroup 4		0.62	(0.14, 2.68)	1.00	(0.87, 1.15)
2013xGroup 2		0.06	(0, 5.61)	1.05	(0.91, 1.21)
2013xGroup 3		-^b^	-	0.99	(0.86, 1.13)
2013xGroup 4		0.39	(0.10, 1.58)	0.95	(0.83, 1.10)
2014xGroup 2		0.10	(0, 3.22)	0.98	(0.85, 1.12)
2014xGroup 3		-^b^	-	0.97	(0.84, 1.12)
2014xGroup 4		0.51	(0.13, 2.05)	0.91	(0.79, 1.05)
2015xGroup 2		0.00	-	1.11	(0.97, 1.27)
2015xGroup 3		-^b^	-	1.04	(0.90, 1.19)
2015xGroup 4		1.07	(0.25, 4.61)	1.03	(0.90, 1.19)
2016xGroup 2		-^b^	-	1.10	(0.95, 1.26)
2016xGroup 3		-^b^	-	1.14*	**(1.00, 1.32)**
2016xGroup 4		1.08	(0.24, 4.99)	1.04	(0.91, 1.20)
**Population (logarithm)**		1.10	(0.85, 1.43)	2.71*	**(2.67, 2.76)**
**Age group (0–4 years as reference)**					
5–9 years	0.80		(0.06, 11.11)	0.74	(0.45, 1.22)
10–14 years	0.18		(0.01, 4.44)	3.84*	**(2.65, 5.55)**
15–19 years	0.12		(0, 96.14)	0.98	(0.63, 1.50)
20–24 years	0.14		(0, 24.45)	0.96	(0.64, 1.45)
25–29 years	0.60		(0.07, 5.16)	1.47	(1.00, 2.17)
30–34 years	0.56		(0.09, 3.50)	3.23*	**(2.27, 4.60)**
35–39 years	0.44		(0.08, 2.54)	5.76*	**(4.09, 8.09)**
40–44 years	0.39		(0.07, 2.17)	12.53*	**(8.96, 17.51)**
45–49 years	0.32		(0.06, 1.68)	22.20*	**(15.93, 30.93)**
50–54 years	0.30		(0.06, 1.58)	35.00*	**(25.15, 48.71)**
55–59 years	0.19*		**(0.04, 0.98)**	51.38*	**(36.95, 71.45)**
60–64 years	0.17*		**(0.03, 0.92)**	74.38*	**(53.49, 103.42)**
65–69 years	0.15*		**(0.03, 0.79)**	89.66*	**(64.50, 124.65)**
70–74 years	0.19*		**(0.04, 0.99)**	167.18*	**(120.24, 232.43)**
75–79 years	0.16*		**(0.03, 0.82)**	244.87*	**(176.18, 340.35)**
80–84 years	0.11*		**(0.02, 0.56)**	322.68*	**(232.17, 448.47)**
85 + years	0.11*		**(0.02, 0.58)**	394.70*	**(284, 548.55)**
**Sex (male as reference)**	0.56*		**(0.40, 0.77)**	0.59*	**(0.58, 0.61)**

^*^P < 0.05

^a^*IRR* Incidence relative risk

^b^These cells were omitted due to large standard error

**Table 7:** Age- and sex-adjusted cancer mortality rate and excess death in areas with different social deprivation level

Year	Group 1 (least deprived)	Group 2	Group 3	Group 4 (most deprived)
2016 Population^a^	985,130	2,253,850	2,118,518	1,977,886
Age & sex adjusted cancer mortality rate (per 100,000 people)				
2011	153.4	162.8	205.5	203.2
2012	145.6	165.5	205.8	203.0
2013	139.9	172.8	210.5	193.2
2014	144.3	163.2	217.4	194.0
2015	139.7	169.7	211.1	195.9
2016	134.8	161.0	222.4	194.9
Excess death^b^ due to social deprivation				
2011	-	211	1103	984
2012	-	448	1276	1135
2013	-	743	1496	1055
2014	-	426	1549	982
2015	-	676	1513	1112
2016	-	592	1856	1189

^a^Population data by large tertiary planning unit groups (LTPUGs) were only available in 2016

^b^Excess death was calculated as the potential reduction in number of death from cancer if the mortality rate drops to Group 1 level in corresponding years

## Discussion

This study updated the Social Deprivation Index for chronic diseases by examining and comparing the validity and robustness of a new SDI (i.e. SDI6) and the previous SDI by Wong et al. [34]. This estimation can be obtained easily since only routine data or statistics are required for its construction. The variables included in the final selected SDI were different from the previous ones [34]. The variables of unemployment, never-married, and one-person household in the previous SDI were replaced by non-managerial occupation, divorce/separate, and two-person household in the present SDI. These changes were mainly data-driven and based on the correlations between the variables and health outcomes. In the factor analysis of the 14 socio-economic variables, it was found that all the six variables used for constructing the SDI had high factor loadings (> 0.5) on factor 1 (f1, explaining 50.9% variance) and factor 2 (f2, explaining 16.9% variance). This showed that the six selected variables are good representation of the socio-economic status. Among them, selection of non-managerial occupation might suggest that lower income and benefits from work, lower status of occupation as well as worse working conditions might also worsen the health condition or healthcare accessibility, as manual laborers earn a salary half to three-fourth that of what managers and professionals earn, but work 20% longer than other occupations in Hong Kong [45]. Removing never-married and one-person household from the SDI might imply a socio-cultural change from 2001. Traditionally, people are expected to get married by certain age, and one of the reasons why some people did not get married by then was believed to be due to poverty or deprivation of resources [46, 47]. However, these factors might not have an effect on health as substantially as a decade ago due to apparently reduced influence of this traditional culture – the proportion of unmarried people aged 25–29 in Hong Kong increased from 75.5 to 83.2% during 2001 to 2016, and those with higher education level had higher unmarried rate, suggesting that the unmarried status might not be due to lower socio-economic status [48]. Household size indicators, including one-, two-, and 6 + person household, were used as candidate indicators in developing the SDI. It was hypothesized that both smaller and excess household size might be associated with worse health outcomes, as small household size were found to be less connected to their community [49] and have more difficulty in receiving adequate peer support for obtaining health information and care, while large household size that is associated with high household dependency ratio [50, 51] might also lead to higher monetary or time costs in seeking healthcare services. Based on the results, one-person and two-person household size was associated with worse health outcomes, but 6 + persons household was associated with better outcomes, which supports the findings in a previous study [49] and suggested that health-related deprivation could be reduced as household size and level of connection to the community increased. Meanwhile, it was more difficult and complicated to explain why two-person household contributed to poorer health outcomes, as it reflects various types of family composition, such as those with two young couples, two older couples, or a-single-parent-with-one-child family. The latter two types might not be able to receive sufficient social support and adequately access healthcare. Sub-tenancy was removed, as there was plenty of missing data on sub-tenancy at LTPUG level, which affected its statistical association with health outcomes, and the missing values also hindered policymakers from obtaining sufficient information when using the SDI.

Our finding was also consistent with previous studies on similar topics. It was found in Hsu et al. [33] that suicide rate in small areas of Hong Kong was associated with lower income, lower education, non-professional occupation, being divorced/separate, single-person households, which was similar to the SDI developed in this study. Some other variables included in Hsu et al., such as Gini index, unemployment rate, unmarried adults, were either unavailable in the latest census dataset or excluded based on validation results in this study. An area-level deprivation index in a South Korean study [20] similar to our newly developed SDI was also constructed using indicators of household size, home ownership, education level, occupation, employment, marital status, and age in order to determine the association between deprivation and cancer survival. The similarity between the two indices suggested that the indicators used for their construction may be potentially generalizable to other high-income East-Asian regions in determining cancer-related deprivation. It also suggested that the SDI developed in this study has a good content validity in measuring deprivation level. Other studies have also found that smaller household size and non-nuclear family were associated with multiple chronic conditions [52, 53], and both household size and income contributed to inequalities in terms of catastrophic health expenditure among chronic disease patients [54]. Education and occupation were also important socio-economic factors affecting accessibility to healthcare [55]. It was also found that divorce was a factor associated with long-term illness among both male and female, while being never-married did not have significant association with long-term illness [6]. These consistent findings suggested a good content validity of our new SDI. Meanwhile, some variables for constructing the index in previous studies, such as ethnicity, amenities of house and car possession [8, 13, 56], were not widely available in routinely collected local dataset at small area level, so these variables were not considered in this study as they would compromise ability to update the index in a timely manner. In our analysis, it was also found that the SDI score was less correlated with all-cause and chronic disease mortality in more recent years. In 2011, the mortality rate in SDI Q3 areas (i.e. second most deprived area) and Q4 areas (i.e. most deprived area) were similar, while there was an expanding gap of the mortality between Q3 and Q4 areas during 2011–2016 (Supplementary file, Figures S3 and S4). This makes the mortality in Q3 become even higher than that in Q4, which could lead to a decreasing correlation between deprivation level and mortality through the years, although the mortality disparity between Q1-Q2 areas and Q3-Q4 areas was still substantial.

There are a number of ways to construct an index. Standardization of variables for constructing the index could be an alternative when these variables vary widely or have different distributions [57]. However, this was not the case in this study using the socio-economic variables from the census data in different small areas, in which standardization of the variables may render the index less comparable across time as standardized index can only reflect relative variation from the population average at certain time points. Another type of index is weighted sum scores, which add weightings to different factors based on factor analysis. It was tested as one of the candidate SDIs in this study, but the result did not show a better criterion validity. A previous study also suggested that the weighting might not be an accurate representation of the relative importance of the factors [57]. Moreover, a single cut-off could be applied to the index for the purpose of better interpretation and reducing uncertainty [41], but the selection of cut-off requires a gold standard for the target measurements, which might not exist. As an alternative, our new SDI used quartiles as relative cut-offs to divide the index, which has been used in several other studies [13, 56, 58].

Our new SDI was then used to determine the association of cancer mortality with social deprivation. It was found that social deprivation was associated with higher cancer mortality in small areas, and the association became stronger in 2016 for those in the second most deprived areas, which suggested deprivation’s impact to cancer mortality was substantial and continuously expanding. This expanding trend of inequalities in cancer mortality was also similar to what previous studies have observed in Western countries regarding cancer incidence, death, and survival [17–19].

In addition to the expanding inequalities, the age- and sex-adjusted cancer mortality rate was increasing dramatically in both the second and third most deprived areas, while the adjusted cancer mortality rate was found to be similar between the most and the second deprived areas, and its increasing trend was even steeper in the second deprived areas than the most deprived ones. These findings may partially result from social security benefits provided by the government as a safety net to those who cannot support themselves financially, namely the Comprehensive Social Security Assistance (CSSA), along with other benefits from non-governmental organizations to these people. Recipients of CSSA make up roughly 3% of population in Hong Kong and are usually considered to be the most financially vulnerable population [59]. CSSA provides a package of benefits including cash allowance and reimbursement to cover medical expenses at public hospitals or clinics [59], which facilitates the receipt of timely and affordable healthcare services. Therefore, this benefit may help people in the most deprived areas and reduce the deprivation’s contribution to cancer mortality. However, not all people lacking in medical access were eligible to receive CSSA, as its eligibility were menas-tested [60]. Those whose income and assets were slightly above the threshold were also likely to suffer from deprivation; however, they are not eligible to receive financial assistance from the government, which might also lead to poorer health outcomes. Therefore, attention and efforts in reducing social deprivation and improving accessibility and affordability of healthcare services should not only be targeted to the most deprived areas and individuals, but a wider range of efforts in communities in addition to the safety net is also needed for prevention and screening of cancer and follow-up of cancer patients. A progressive financial subsidies scheme which also covers those with financial status above the current CSSA cut-off can be considered in replacement of a single cut-off threshold of income and assets. In other words, the concept of socio-economic gradient in health should be taken into account while formulating relevant policies.

### Limitations

Firstly, this is an ecological study where ecological fallacy might occur as it used area-level data for examination of association instead of individual-level information. Although the variables used were consistent with studies at individual level, the effect size of SDI on health outcomes might differ. However, this study provides evidence that area-level social deprivation could be a way to monitor social deprivation patterns, which is easier to implement than using individual-level SDI that requires collection of individual-level data. Secondly, inpatient episode in public hospital system was used as a morbidity outcome in the study; however, higher inpatient episode could represent either higher morbidity or higher accessibility to healthcare service, or both, which could be one of the reasons why non-fatal outcomes were not used as often as mortality in previous assessment of disease burden [61]. Using public hospitals inpatient episode as morbidity indicator may underestimate the actual chronic disease morbidity of deprived individuals as they might have less access to inpatient services [62]. Affluent persons may also be less likely to use public service as they can afford private inpatient services. However, public hospitals provide up to 90% of inpatient bed days in Hong Kong [63], which means the gaps between overall and public inpatient service utilization, as well as the bias caused by private inpatient service utilization, are relatively small. Considering this, methods to determine chronic disease prevalence from more comprehensive sources that capture public and private service utilization and unmet needs should be explored in future studies. Nevertheless, this study used mortality indicators in addition to the public inpatient services utilization as health outcome indicators.. Thirdly, the exclusion of inpatient episode without residential area information (i.e. missing data) may lead to an underestimation of chronic disease morbidity, which tended to be greater in more deprived areas (SDI Q3 and Q4 areas, see Supplementary file). Considering the higher chronic disease morbidity found in more deprived areas using existing data (Figure S2), the gaps of morbidity across areas with different deprivation levels could be even larger and the correlation between morbidity and social deprivation could be stronger if the missing data were taken into account. Lastly, although the factor analysis and similarity between our SDI and other previously developed deprivation indices suggested good representativeness of the variables in measuring socio-economic status, their associations with deprivation level were not directly examined as there is no gold standard for measuring social deprivation. Nevertheless, according to a framework adopted by the British government in measuring social deprivation [64], the variables used to construct their SDI included the domains of income (weight = 22.5%), employment (weight = 22.5%), education (weight = 13.5%), health (weight = 13.5%), barriers to housing and service (weight = 9.3%), crime (weight = 9.3%), and living environment (weight = 9.3%). These are similar to the variables used in constructing our SDI. The domain of health was not included in our SDI to avoid duplicates with the outcomes of this study. The domains of crime and living environment were also not used to construct our SDI since they were not available in Hong Kong’s Census data; however, they had relatively lower weighting in the overall measurement. While this framework showed that these routinely available variables can be used to measure deprivation level, Delphi panel study can be conducted in future studies for further confirmation.

## Conclusions

The updated SDI is a valid measurement of social deprivation at small areas and is useful in health planning, resource allocation, and design for community public health strategies and interventions. The analysis of SDI and cancer mortality demonstrated the usage of the index, and found an expanding health inequality in cancer outcomes among small areas with different deprivation level through the past few years. It highlighted the importance to take the concepts of deprivation and socio-economic gradient in health into account in devising policies and interventions in order to tackle and reduce health inequalities, and expand the coverage of policies and interventions for alleviating health-related deprivation to a wider range of population and areas. Future works can be conducted to improve implementation of the index and design follow-up strategies for improvement of health and healthcare services in deprived areas.

## Supplementary Information

**Additional file 1.** ICD-10 codes for chronic disease selection, characteristics of missing data, mortality and morbidity according to deprivation level during 2011-2015/2016, and geographical variation of social deprivation and cancer mortality.

## Data Availability

The data related to socio-economic characteristics of Hong Kong small areas are available on website of Hong Kong 2016 By-Census (https://www.bycensus2016.gov.hk/en/). The rest of data analyzed in this study are not publicly available as they are confidential. These data need to be obtained from Hospital Authority of Hong Kong.

## Abbreviations

CI: Confidence interval
COPD: Chronic obstructive pulmonary disease
CSSA: Comprehensive Social Security Assistance
ICD: International Classification of Diseases
IRR: Incidence relative risk
LTPUG: Large tertiary planning unit groups
SDI: Social deprivation index;
SER: Standardized episode rate
SMR: Standardized mortality rate
UPA score: Underprivileged Area score

## References

[1] Lamnisos D, Lambrianidou G, Middleton N (2019). Small-area socioeconomic deprivation indices in Cyprus: development and association with premature mortality. BMC Public Health.

[2] Townsend P, Phillimore P, Beattie A. Health and deprivation: inequality and the North. UK: Routledge; 1988.

[3] Carstairs V, Morris R (1989). Deprivation: explaining differences in mortality between Scotland and England and Wales. BMJ.

[4] Jarman B (1983). Identification of underprivileged areas. Br Med J (Clin Res Ed).

[5] Chateau D, Metge C, Prior H, Soodeen R-A. Learning from the census: the Socio-economic Factor Index (SEFI) and health outcomes in Manitoba. Can J Public Health. 2012;103(Suppl 2):S23–S27. 10.1007/BF03403825 PMC6973861 23618067

[6] Robards J, Evandrou M, Falkingham J, Vlachantoni A (2012). Marital status, health and mortality. Maturitas.

[7] Dolan SA, Jarman B, Bajekal M, Davies PM, Hart D (1995). Measuring disadvantage: changes in the underprivileged area, Townsend, and Carstairs scores 1981–91. J Epidemiol Community Health.

[8] Guillaume E, Pornet C, Dejardin O, Launay L, Lillini R, Vercelli M (2016). Development of a cross-cultural deprivation index in five European countries. J Epidemiol Community Health.

[9] Benach J, Yasui Y (1999). Geographical patterns of excess mortality in Spain explained by two indices of deprivation. J Epidemiol Community Health.

[10] Eibner C, Sturm R (2006). US-based indices of area-level deprivation: results from HealthCare for Communities. Soc Sci Med.

[11] Frohlich N, Mustard C (1996). A regional comparison of socioeconomic and health indices in a Canadian province. Soc Sci Med.

[12] Salmond C, Crampton P, Sutton F (1998). NZDep91: a New Zealand index of deprivation. Aust N Z J Public Health.

[13] Singh GK (2003). Area deprivation and widening inequalities in US mortality, 1969–1998. Am J Public Health.

[14] Singh GK, Siahpush M (2006). Widening socioeconomic inequalities in US life expectancy, 1980–2000. Int J Epidemiol.

[15] Singh GK, Azuine RE, Siahpush M, Kogan MD (2013). All-cause and cause-specific mortality among US youth: socioeconomic and rural-urban disparities and international patterns. J Urban Health.

[16] Mackenbach JP, Bos V, Andersen O, Cardano M, Costa G, Harding S (2003). Widening socioeconomic inequalities in mortality in six Western European countries. Int J Epidemiol.

[17] Tweed EJ, Allardice GM, McLoone P, Morrison DS (2018). Socio-economic inequalities in the incidence of four common cancers: a population-based registry study. Public Health.

[18] Teng AM, Atkinson J, Disney G, Wilson N, Blakely T (2017). Changing socioeconomic inequalities in cancer incidence and mortality: cohort study with 54 million person-years follow-up 1981–2011. Int J Cancer.

[19] Dalton SO, Olsen MH, Johansen C, Olsen JH, Andersen KK (2019). Socioeconomic inequality in cancer survival - changes over time. A population-based study, Denmark, 1987–2013. Acta Oncol.

[20] Kwak M, Kim C (2018). Effect of area-level deprivation on cancer survival time: a register-based follow-up study of 145 585 Korean subjects. Asia Pac J Public Health.

[21] Ha M, Hwang SS, Kang S, Park NW, Chang BU, Kim Y (2017). Geographical correlations between indoor radon concentration and risks of lung cancer, non-Hodgkin’s lymphoma, and leukemia during 1999–2008 in Korea. Int J Environ Res Public Health.

[22] World Bank. World Bank Open Data 2019. Available from: https://data.worldbank.org/country/XD. Accessed 11 Dec 2020.

[23] Social, IoHK. Gini index 2017. Available from: https://www.socialindicators.org.hk/en/indicators/economy/11. Accessed 10 Dec 2020.

[24] Census and Statistics Department. Hong Kong population projections 2017–2066.Hong Kong; 2017. Available from: https://www.statistics.gov.hk/pub/B1120015072017XXXXB0100.pdf.

[25] Census and Statistics Department. Thematic household survey report no. 40. Hong Kong; 2009. Available from: https://www.statistics.gov.hk/pub/B11302402009XXXXB0100.pdf.

[26] Census and Statistics Department. Thematic household survey report no. 68. Hong Kong; 2019 Available from: https://www.statistics.gov.hk/pub/B11302682019XXXXB0100.pdf.

[27] Roth GA, Abate D, Abate KH, Abay SM, Abbafati C, Abbasi N (2018). Global, regional, and national age-sex-specific mortality for 282 causes of death in 195 countries and territories, 1980–2017: a systematic analysis for the Global Burden of Disease Study 2017. Lancet.

[28] Centre for Health Protection. Death rates by leading causes of death, 2001 – 2019. Hong Kong; 2020 Available from: https://www.chp.gov.hk/en/statistics/data/10/27/117.html.

[29] Chung GK, Lai FT, Chan DC, Wong H, Yeoh EK, Chung RY (2020). Socioeconomic disadvantages over the life-course and their influence on obesity among older Hong Kong Chinese adults. Eur J Public Health.

[30] Chung GKK, Chung RYN, Chan DCC, Lai FTT, Wong H, Lau MKW (2019). The independent role of deprivation in abdominal obesity beyond income poverty. A population-based household survey in Chinese adults. J Public Health.

[31] Chung RYN, Chung GKK, Gordon D, Wong SYS, Chan D, Lau MKW (2018). Deprivation is associated with worse physical and mental health beyond income poverty: a population-based household survey among Chinese adults. Qual Life Res.

[32] Chung RYN, Marmot M, Mak JKL, Gordon D, Chan D, Chung GKK (2021). Deprivation is associated with anxiety and stress. A population-based longitudinal household survey among Chinese adults in Hong Kong. J Epidemiol Community Health.

[33] Hsu C-Y, Chang S-S, Lee ES, Yip PS (2015). Geography of suicide in Hong Kong: spatial patterning, and socioeconomic correlates and inequalities. Soc Sci Med.

[34] Wong CM, Ou CQ, Chan KP, Chau YK, Thach TQ, Yang L (2008). The effects of air pollution on mortality in socially deprived urban areas in Hong Kong, China. Environ Health Perspect.

[35] Low CT, Lai PC, Li HD, Ho WK, Wong P, Chen S (2016). Neighbourhood effects on body constitution–a case study of Hong Kong. Soc Sci Med.

[36] Sun S, Laden F, Hart JE, Qiu H, Wang Y, Wong CM (2018). Seasonal temperature variability and emergency hospital admissions for respiratory diseases: a population-based cohort study. Thorax.

[37] Wong PP-Y, Lai PC, Low CT, Chen S, Hart M (2016). The impact of environmental and human factors on urban heat and microclimate variability. Build Environ.

[38] Thach TQ, Zheng Q, Lai PC, Wong PPY, Chau PYK, Jahn HJ (2015). Assessing spatial associations between thermal stress and mortality in Hong Kong: a small-area ecological study. Sci Total Environ.

[39] Li VO, Han Y, Lam JC, Zhu Y, Bacon-Shone J (2018). Air pollution and environmental injustice: are the socially deprived exposed to more PM2.5 pollution in Hong Kong?. Environ Sci Policy.

[40] Census and Statistics Department. 2016 population by-census. Hong Kong; 2016. Available from: https://www.bycensus2016.gov.hk/en/.

[41] Allik M, Leyland A, Travassos Ichihara MY, Dundas R (2020). Creating small-area deprivation indices: a guide for stages and options. J Epidemiol Community Health.

[42] Census and Statistics Department. Thematic household survey report no. 50. Hong Kong; 2013. Available from: https://www.statistics.gov.hk/pub/B11302502013XXXXB0100.pdf.

[43] Census and Statistics Department. Hong Kong poverty situation report 2012. Hong Kong; 2013. Available from: https://www.povertyrelief.gov.hk/pdf/2012_Poverty_Situation_Eng.pdf.

[44] Yang S (2014). A comparison of different methods of zero-inflated data analysis and its application in health surveys.

[45] Census and Statistics Department. 2019 report on annual earnings and hours survey. Hong Kong; 2019. Available from: https://www.statistics.gov.hk/pub/B10500142019AN19B0100.pdf.

[46] Gao Q, Garfinkel I, Zhai F (2009). Anti-poverty effectiveness of the minimum living standard assistance policy in urban China. Rev Income Wealth.

[47] Zhou XD, Wang XL, Li L, Hesketh T (2011). The very high sex ratio in rural China: impact on the psychosocial wellbeing of unmarried men. Soc Sci Med.

[48] Census and Statistics Department. Marriage and divorce trends in Hong Kong, 1991 to 2016. Hong Kong; 2018. Available from: https://www.statistics.gov.hk/pub/B71801FB2018XXXXB0100.pdf.

[49] Wong H, Saunders P, Wong WP, Chan M, Chua HW (2012). Final report of research study on the deprivation and social exclusion in Hong Kong.

[50] Hadley C, Belachew T, Lindstrom D, Tessema F (2011). The shape of things to come? household dependency ratio and adolescent nutritional status in rural and urban Ethiopia. Am J Phys Anthropol.

[51] Kelley AC (1973). Population growth, the dependency rate, and the pace of economic development. Popul Stud.

[52] Hopman P, Schellevis FG, Rijken M (2016). Health-related needs of people with multiple chronic diseases: differences and underlying factors. Qual Life Res.

[53] Park E-J, Sohn HS, Lee EK, Kwon JW (2014). Living arrangements, chronic diseases, and prescription drug expenditures among Korean elderly: vulnerability to potential medication underuse. BMC Public Health.

[54] Wang Z, Li X, Chen M (2015). Catastrophic health expenditures and its inequality in elderly households with chronic disease patients in China. Int J Equity Health.

[55] Elwell-Sutton TM, Jiang CQ, Zhang WS, Cheng KK, Lam TH, Leung GM (2013). Inequality and inequity in access to health care and treatment for chronic conditions in China: the Guangzhou Biobank Cohort Study. Health Policy Plan.

[56] Smith JJ, Tilney HS, Heriot AG, Darzi AW, Forbes H, Thompson MR (2006). Social deprivation and outcomes in colorectal cancer. Br J Surg.

[57] DiStefano C, Zhu M, Mindrila D (2009). Understanding and using factor scores: Considerations for the applied researcher. Pract Assess Res Eval.

[58] ELHadi A, Ashford-Wilson S, Brown S, Pal A, Lal R, Aryal K (2016). Effect of social deprivation on the stage and mode of presentation of colorectal cancer. Ann Coloproctol.

[59] Census and Statistics Department. Statistics on comprehensive social security assistance scheme, 2007 to 2017. Hong Kong; 2018. Available from: https://www.statistics.gov.hk/pub/B71809FB2018XXXXB0100.pdf.

[60] Wong SYS, Chung RYN, Chan D, Chung GKK, Li J, Mak D (2018). What are the financial barriers to medical care among the poor, the sick and the disabled in the Special Administrative Region of China?. PloS One.

[61] Thacker SB, Stroup DF, Carande-Kulis V, Marks JS, Roy K, Gerberding JL (2006). Measuring the public’s health. Public Health Rep.

[62] Wong C, Hedley A, Lam T, Bacon-Shone J (1999). Estimation of variations in health care needs between small areas in HK using routine statistics on morbidity, mortality and socioeconomic and demographic characteristics of the population. Health, Welfare and Food Bureau, Hong Kong.

[63] Schoeb V (2016). Healthcare service in Hong Kong and its challenges the role of health professionals within a social model of health. China Perspect.

[64] Ministry of Housing, Communities & Local Government. The English Indices of Deprivation 2019 (IoD2019). UK; 2019. Available from: https://assets.publishing.service.gov.uk/government/uploads/system/uploads/attachment_data/file/835115/IoD2019_Statistical_Release.pdf.

