# Supplementary material for: Measuring health-related social deprivation in small areas: development of an index and examination of its association with cancer mortality
Source: Int J Equity Health. 2021 Sep 27;20:216. doi: 10.1186/s12939-021-01545-9 (PMC8474923; doi:10.1186/s12939-021-01545-9)
Supplement: Supplementary file 1 — Additional file 1. ICD-10 codes for chronic disease selection, characteristics of missing data, mortality and morbidity according to deprivation level during 2011-2015/2016, and geographical variation of social deprivation and cancer mortality. [file 12939_2021_1545_MOESM1_ESM.docx]

**Supplementary file.**

1. ICD-10 codes for chronic disease selection.

The International Statistical Classification of Diseases and Related Health Problems 10th Revision (ICD-10) was used to select chronic diseases for analysis. The ICD-10 codes of selected diseases are listed in Table A1.

Table S1. ICD-10 codes of selected chronic diseases

| **Disease or condition** | **ICD-10 codes** |
| --- | --- |
| Hypertension | I10 |
| Diabetes mellitus | E10, E11, E14 |
| High cholesterol | E78 |
| Heart diseases | I20-I25, I27-I28, I30, I31, I33-I39, I40, I42, I44, I45, I49, I50, I51 |
| Asthma | J45 |
| Chronic obstructive pulmonary disease (COPD) | J40-J44, J47 |
| Stroke or cerebrovascular diseases | I60-I67, I69, G45 |
| Cancer or neoplasms | C00-C96, D00-D49 |

1. Characteristics of missing data

In the Social Deprivation Index (SDI) development, the inpatient episodes without residence area were removed from the analysis. The characteristics of the missing episodes were described and summarized in below tables in comparison with the episodes included in the analysis (Table S2). The overall percentage of missing episodes was 26.5%. It was found that there were more episodes with missing data (i.e. excluded episodes) among those with advanced age (age ≥ 75 years, 22.9%-49.5% vs 12.5-18.5% in age between 0-74), male (30.3% vs 23.4% in female), and patients with stroke/cardiovascular conditions (35.6% vs 24.3% for those without the condition), and those without asthma (26.9% vs 19.4% for those with the condition) or high cholesterol (26.6% vs 20.7% for those with the condition). On the other hand, the percentage of episodes with missing data was similar across patients with different age between 0-74 years, and patients with or without hypertension, diabetes, heart disease, and COPD.

These disproportionate distributions across the aforementioned characteristics could lead to a greater underestimate of inpatient episode in areas with more older persons aged 75+ years, more male, more stroke/cardiovascular conditions, and less Asthma and high cholesterol. It was found that the more deprived areas (SDI Q3 and Q4 areas) tended to have more persons aged 75+ years and slightly more male (Table S3), as well as higher stroke/cardiovascular mortality (2011-2016) than less deprived areas (SDI Q1 and Q2 areas) (Figure S1), while there was no difference in asthma and high cholesterol mortality, so the inpatient episode were more likely to be underestimated in more deprived areas. Considering the higher chronic disease morbidity found in more deprived areas using the existing data (Figure S2), the gaps of morbidity across areas could be even larger and the correlation between morbidity and social deprivation could be stronger if the missing data were taken into account.

Table S2. The characteristics of the episode excluded from the analysis due to missing data.

|  | Excluded episode [N(%)] | Included episode [N(%)] | Overall [N(%)] |
| --- | --- | --- | --- |
| **Age (years)** | |  |  |
| 0-4 | 4502 (15.8) | 24075 (84.3) | 28577 (100.0) |
| 5-9 | 1910 (14.1) | 11669 (85.9) | 13579 (100.0) |
| 10-14 | 1323 (13.0) | 8854 (87.0) | 10177 (100.0) |
| 15-19 | 1359 (12.5) | 9556 (87.6) | 10915 (100.0) |
| 20-24 | 1392 (13.8) | 8721 (86.2) | 10113 (100.0) |
| 25-29 | 1948 (13.8) | 12214 (86.2) | 14162 (100.0) |
| 30-34 | 3397 (16.1) | 17746 (83.9) | 21143 (100.0) |
| 35-39 | 4962 (16.6) | 24866 (83.4) | 29828 (100.0) |
| 40-44 | 8190 (17.4) | 38935 (82.6) | 47125 (100.0) |
| 45-49 | 13171 (15.7) | 71007 (84.4) | 84178 (100.0) |
| 50-54 | 21037 (15.5) | 114358 (84.5) | 135395 (100.0) |
| 55-59 | 28324 (15.3) | 157058 (84.7) | 185382 (100.0) |
| 60-64 | 34806 (15.4) | 191257 (84.6) | 226063 (100.0) |
| 65-69 | 41825 (16.3) | 215407 (83.7) | 257232 (100.0) |
| 70-74 | 64469 (18.5) | 283623 (81.5) | 348092 (100.0) |
| 75-79 | 110180 (22.9) | 370421 (77.1) | 480601 (100.0) |
| 80-84 | 153250 (30.6) | 348381 (69.5) | 501631 (100.0) |
| 85+ | 306319 (49.5) | 312468 (50.5) | 618787 (100.0) |
| **Sex** |  |  |  |
| Male | 416912 (30.3) | 960287 (69.7) | 1377199 (100.0) |
| Female | 385452 (23.4) | 1260329 (76.6) | 1645781 (100.0) |
| **Hypertension** | |  |  |
| No | 493468 (25.9) | 1413625 (74.1) | 1907093 (100.0) |
| Yes | 308896 (27.7) | 806991 (72.3) | 1115887 (100.0) |
| **Diabetes** |  |  |  |
| No | 581586 (27.2) | 1558501 (72.8) | 2140087 (100.0) |
| Yes | 220778 (25.0) | 662115 (75.0) | 882893 (100.0) |
| **High Cholesterol** | |  |  |
| No | 798111 (26.6) | 2204344 (73.4) | 3002455 (100.0) |
| Yes | 4253 (20.7) | 16272 (79.3) | 20525 (100.0) |
| **Heart disease** | |  |  |
| No | 443986 (26.2) | 1251813 (73.8) | 1695799 (100.0) |
| Yes | 358378 (27.0) | 968803 (73.0) | 1327181 (100.0) |
| **Asthma** |  |  |  |
| No | 775938 (26.9) | 2110580 (73.1) | 2886518 (100.0) |
| Yes | 26426 (19.4) | 110036 (80.6) | 136462 (100.0) |
| **COPD** |  |  |  |
| No | 672994 (26.3) | 1882757 (73.7) | 2555751 (100.0) |
| Yes | 129370 (27.7) | 337859 (72.3) | 467229 (100.0) |
| **Stroke/Cardiovascular disease** | |  |  |
| No | 591624 (24.3) | 1839585 (75.7) | 2431209 (100.0) |
| Yes | 210740 (35.6) | 381031 (64.4) | 591771 (100.0) |
| **Total** | 802364 (26.5) | 2220616 (73.5) | 3022980 (100.0) |

Table S3. Percentage of people aged 75+ years and male at areas with different deprivation level

| SDI quartile | Age 75+ years | Male percentage |
| --- | --- | --- |
| Q1 | 5.4% | 43.5% |
| Q2 | 6.8% | 46.0% |
| Q3 | 7.7% | 46.6% |
| Q4 | 8.9% | 46.7% |
| Total | 7.5% | 46.0% |

Figure S1. Age- and sex-standardized stroke/cardiovascular mortality rate during 2011-2016

Figure S2. Age- and sex-standardized chronic disease inpatient episode rate during 2011-2015

1. Supplementary figure: all-cause and chronic disease mortality according to deprivation level during 2011-2016

Figure S3. Age- and sex-standardized all-cause mortality rate during 2011-2016

Figure S4. Age- and sex-standardized chronic disease mortality rate during 2011-2016

1. Geographical variation of social deprivation levels and cancer mortality in Hong Kong

Figure S5. Geographical variation of social deprivation levels (in SDI quartiles) in Hong Kong


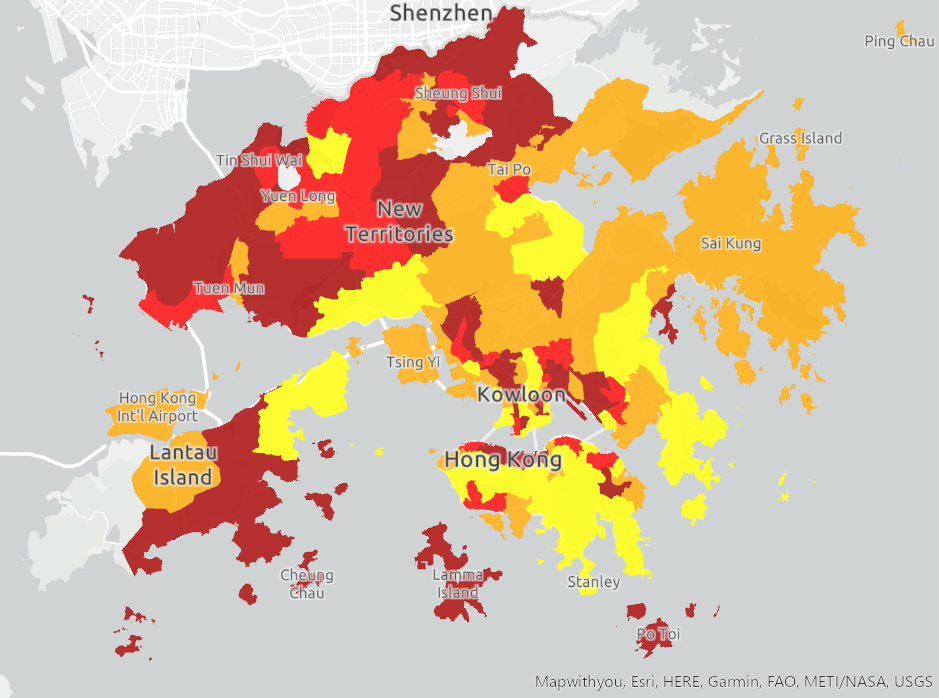


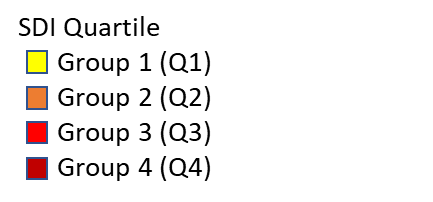


Figure S6. Geographical variation of cancer mortality in Hong Kong


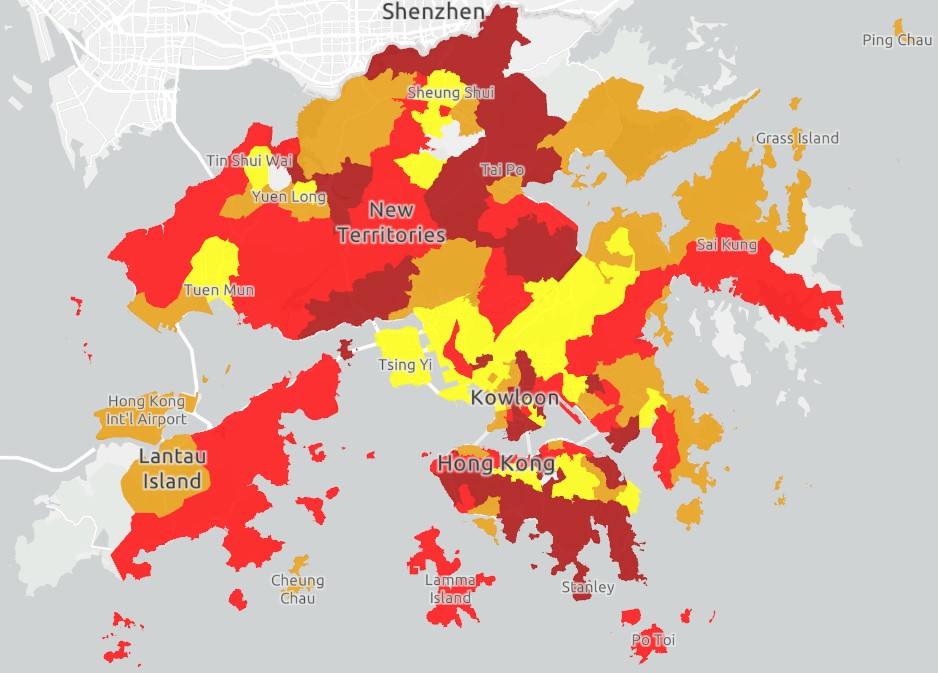


Cancer Mortality Quartile

SMR ≤ 0.34 (Q1)

0.34 < SMR ≤ 0.75 (Q2)

0.75 < SMR ≤ 2.10 (Q3)

SMR > 2.10 (Q4)
